# Correction: CD81^+^ senescent-like fibroblasts exaggerate inflammation and activate neutrophils via C3/C3aR1 axis in periodontitis

**DOI:** 10.7554/eLife.111259

**Published:** 2026-03-13

**Authors:** Liangliang Fu, Chenghu Yin, Qin Zhao, Shuling Guo, Wenjun Shao, Ting Xia, Quan Sun, Liangwen Chen, Jinghan Li, Min Wang, Haibin Xia

**Keywords:** Human, Mouse

Fu L, Yin C, Zhao Q, Guo S, Shao W, Xia T, Sun Q, Chen L, Li J, Wang M, Xia H. 2025. CD81+ senescent-like fibroblasts exaggerate inflammation and activate neutrophils via C3/C3aR1 axis in periodontitis. *eLife* **13**:RP96908. doi: 10.7554/eLife.96908.

Published 13 August 2025

A concern was raised by a reader regarding potential batch effects in the fibroblast subclustering analysis. Specifically, it was noted that potential batch effects after fibroblast subsetting might produce sample-driven clusters. We acknowledge that batch effects were present in the initial subclustering and have been mitigated following integration. In response to batch effects in the fibroblast subclustering, we provide new fibroblast subclusters after batch correction using Harmony and updated Figure 3A and B. Accordingly, senescence scores and CD81^+^ fibroblast identification in Figure 3, SASP related-genes expression heatmap and pseudotemporal trajectory inference in Figure 4 and cell-cell communication inferences in Figure 5 were revised as well after batch correction. Texts and figures of the Figure 4—supplement 1 were removed due to no enrichment of lipid metabolism pathway in CD81^+^ fibroblasts after batch effect removal.

In specific, following the rigorous batch effect removal, the proportionate distribution of fibroblast subclusters between healthy group and periodontitis group is comparable and now aligns with that reported in the original paper (https://doi.org/10.1016/j.cell.2021.05.013). Crucially, the re-analysis still reconfirms the existence of the CD81^+^ senescent-like fibroblast subpopulation and its role in C3-C3aR1 signaling with neutrophils. Although the major conclusions of the article remain unaffected, we are providing the updated bioinformatics results and figures to ensure the highest level of scientific rigor and reproducibility.

This necessitated the changes to the Results, Materials and Methods, and Figure and legends as described below. Underline indicates differences between corrected and original text.

**Results**

**[Corrected text]:**

The cell proportion bar chart revealed an increase in subpopulations 0 in periodontitis compared to healthy controls (Figure 3B). We then applied a cellular senescence gene set (Saul et al., 2022) to score these subpopulations and found that subpopulation 0 exhibited the highest average expression levels, with a marked increase in periodontitis (Figure 3C). Gene Ontology (GO) enrichment analysis of the differentially expressed genes further confirmed that subpopulation 0 displayed upregulated aging characteristics (Figure 3D), indicating that this subpopulation is primarily responsible for fibroblast senescence. A density heatmap demonstrated that CD81 was predominantly enriched in subpopulation 0 (Figure 3E).

**[Original text]:**

The cell proportion bar chart revealed a significant increase in subpopulations 1 and 3 in periodontitis compared to healthy controls (Figure 3B). We then applied a cellular senescence gene set (Saul et al., 2022) to score these subpopulations and found that subpopulation 1 exhibited the highest average expression levels, with a marked increase in periodontitis (Figure 3C). Gene Ontology (GO) enrichment analysis of the differentially expressed genes further confirmed that subpopulation 1 displayed upregulated aging characteristics (Figure 3D), indicating that this subpopulation is primarily responsible for fibroblast senescence. Among the top 20 marker genes for subpopulation 1, CD81, a transmembrane protein, emerged as a potential biomarker for this senescent subpopulation (Figure 3E). A density heatmap demonstrated that CD81 was predominantly enriched in subpopulation 1 (Figure 3F).

**[Removed text]**:

The following text has been removed due to no enrichment of lipid metabolism pathway in CD81 +fibroblasts after batch effect removal:

‘Additionally, we examined the metabolic activity of each subgroup, focusing on lipid metabolism. Pathways related to fatty acid biosynthesis, arachidonic acid metabolism, and steroid biosynthesis were significantly upregulated in CD81^+^ fibroblasts (Figure 4—figure supplement 1A), suggesting that lipid metabolism might play a role in cellular senescence of the gingival fibroblasts. Arachidonic acid, in particular, could be converted into prostaglandins and leukotrienes via cyclooxygenases (COXs) and lipoxygenases, contributing to the inflammatory response (Figure 4—figure supplement 1B; Wang et al., 2021). We further observed a higher gene expression of PTGS1 (encoding COX1 protein) and PTGS2 (encoding COX2 protein) in CD81^+^ fibroblasts compared to other fibroblast subpopulations (Figure 4—figure supplement 1C).’

**[Corrected text]**:

Several SASP genes, including CXCL1, CXCL6, CXCL8, IL6, SERPINE1, IGFBP4, MMP1 and TIMP1, also exhibited increased expression during differentiation (Figure 4E).

**[Original text]**:

Several SASP genes, including CXCL1, CXCL6, IL6, MMP1, SERPINE1, EGFR, FGF2, FNDC1, IGFBP4, LAMB1, and TIMP1, also exhibited increased expression during differentiation (Figure 4E).

**[Corrected text]**:

Additionally, we observed a significant increase in the expression of C3 signaling pairs between CD81^+^ fibroblasts and neutrophil cells (Figure 5B).

**[Original text]**:

Additionally, we observed a significant increase in the expression of MIF and C3 signaling pairs between CD81^+^ fibroblasts and immune cells (Figure 5B).

**Materials and Methods**

**[Added text]** in the method of Fibroblast cell re-clustering analysis:

The following text has been added as the method description of the batch correction to the updated figure:

‘Harmony was applied to correct for inter-sample variation, and the Harmony components were used for clustering and UMAP embedding. ’

**[Removed text]** in the method description of metabolism pathway analysis:

The following text has been removed as the contents are no longer relevant to the updated results:

‘Metabolism pathway analysis.The ‘scMetabolism’ package was used to quantify the metabolism activity at the scRNA-seq dataset. Seventy-eight metabolism pathways in KEGG were included in the package. The pathways were further used to evaluate the metabolism activity at the single-cell resolution (Wu et al., 2022).’

**Figure Legends**

**[Corrected text]:**

Figure 3 (D) Fibroblast subcluster 0 shows enrichment of aging process highlighted by red. (E) Density map of CD81 expression among fibroblast subclusters. (F) Re-annotation of fibroblast subcluster according to GO analysis.

**[Original text:]**

Figure 3 (D) Fibroblast subcluster 1 shows enrichment of aging process highlighted by red. (E) Cellular localization of the top 20 marker molecules in fibroblasts subcluster 1. CD81 protein, located at cell membrane, was highlighted by red. (F) Density map of CD81 expression among fibroblast subcluster and re-annotation of fibroblast subcluster according to GO analysis.

**[Removed text]** in the legends of Figure 4—supplement 1:

The following text has been removed as the contents are no longer relevant to the updated results:

‘Figure 4—supplement 1 Metabolic pathways analysis of fibroblast subclusters. (A) The heatmap representing metabolic pathways in each fibroblast subcluster, which indicated fatty acid biosynthesis, arachidonic acid metabolism, and steroid biosynthesis, was significantly upregulated in CD81^+^ fibroblasts. (B) The flow chart representing the metabolism of arachidonic acid, which could be converted into prostaglandins (PGs) and Thromboxane As (TXAs) by COX-1 or COX-2. (C) The dot plot representing that PTGS1 gene (encoding COX1 protein) and PTGS2 gene (encoding COX2 protein) are significantly higher in CD81^+^ gingival fibroblasts compared to other fibroblast subclusters.’

**Figures**

The corrected Figure 3 panel A-F after batch correction is shown here:

Figure 3E in the previous article was removed due to the fact that CD81 was no longer among the TOP 20 marker genes in the senescence-related fibroblast subcluster 0.

**Figure 1:**
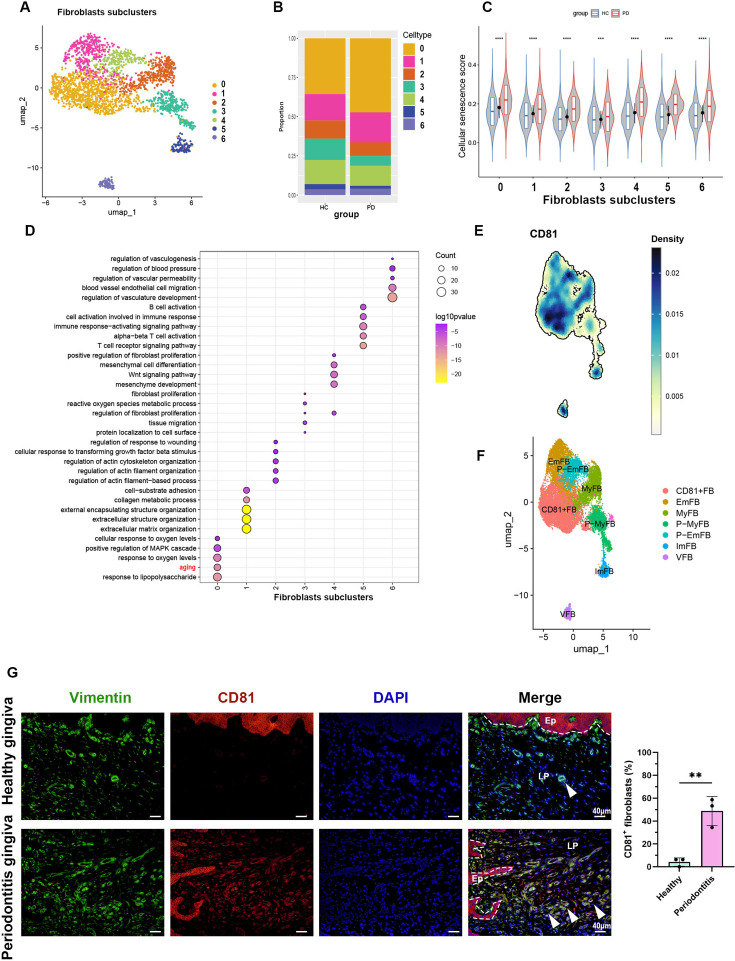


The originally published Figure 3 is shown for reference:

**Figure 2:**
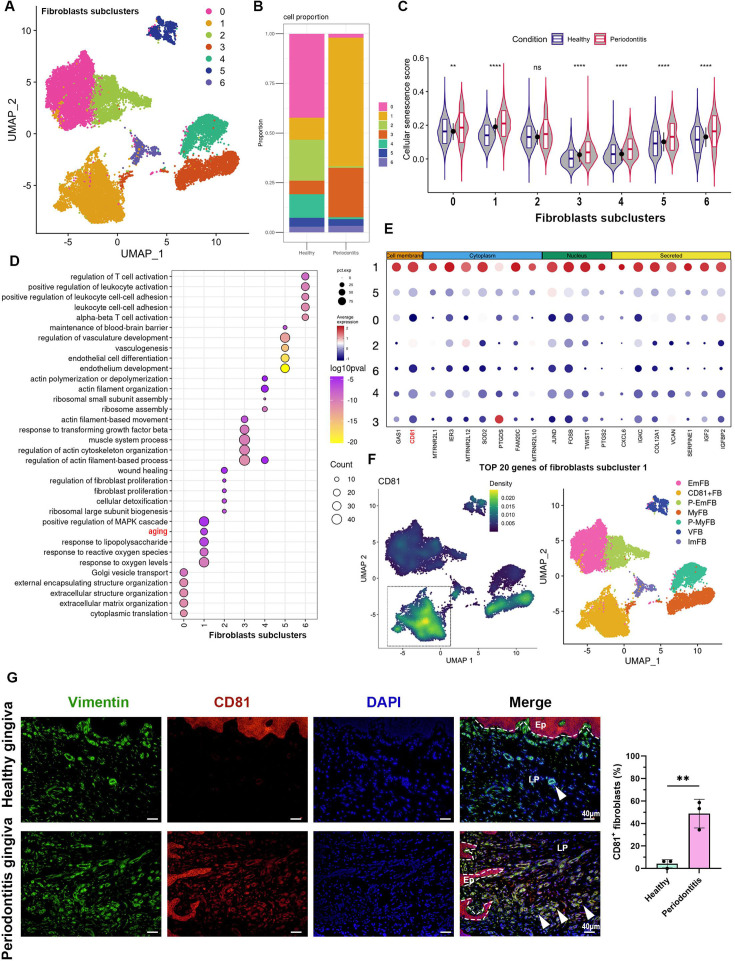


The corrected Figure 4 after batch correction is shown here:

**Figure 3:**
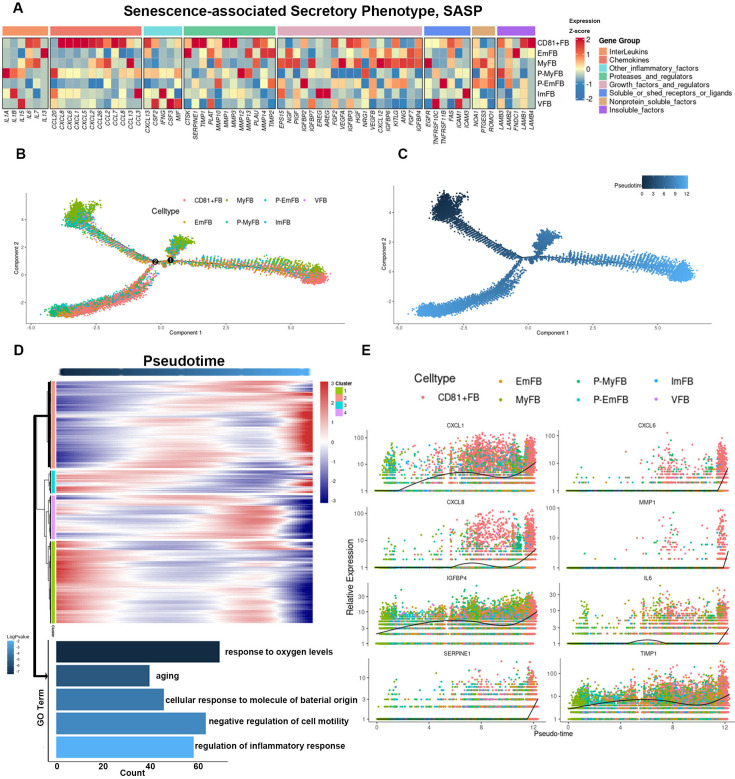


The originally published Figure 4 is shown for reference:

**Figure 4:**
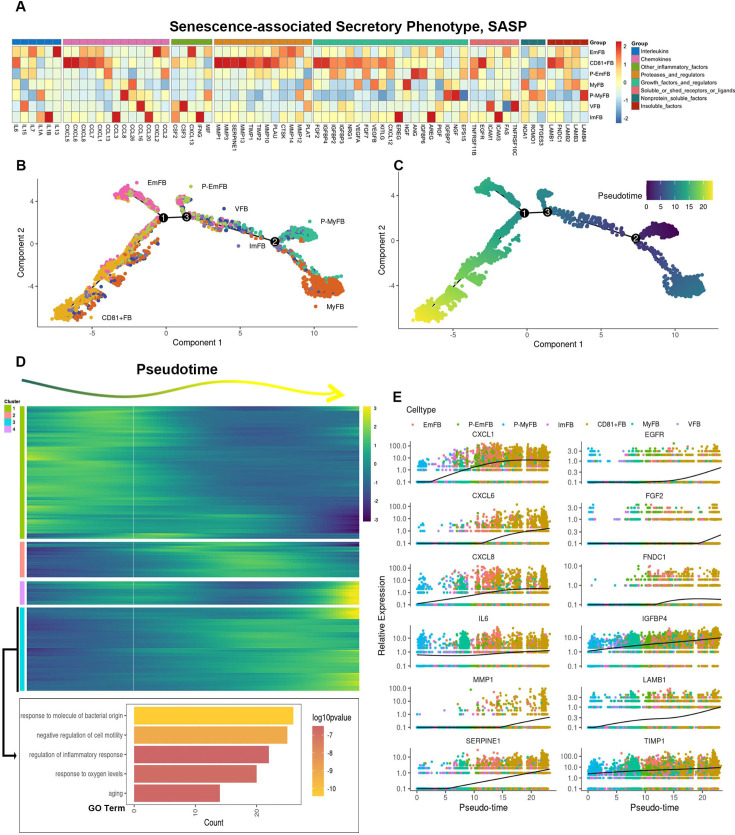


**[Removed Figure 4—figure supplement 1]:**

Figure 4—figure supplement 1 was removed due to no enrichment of lipid metabolism pathway in CD81^+^ fibroblasts after batch effect removal.

**Figure 5:**
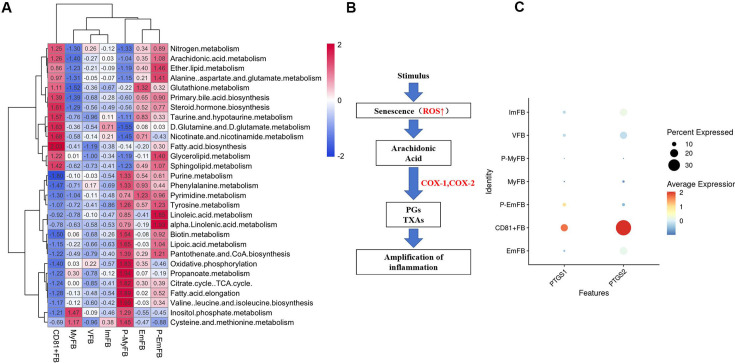


The corrected Figure 5 panel A-D after batch correction is shown here:

**Figure 6:**
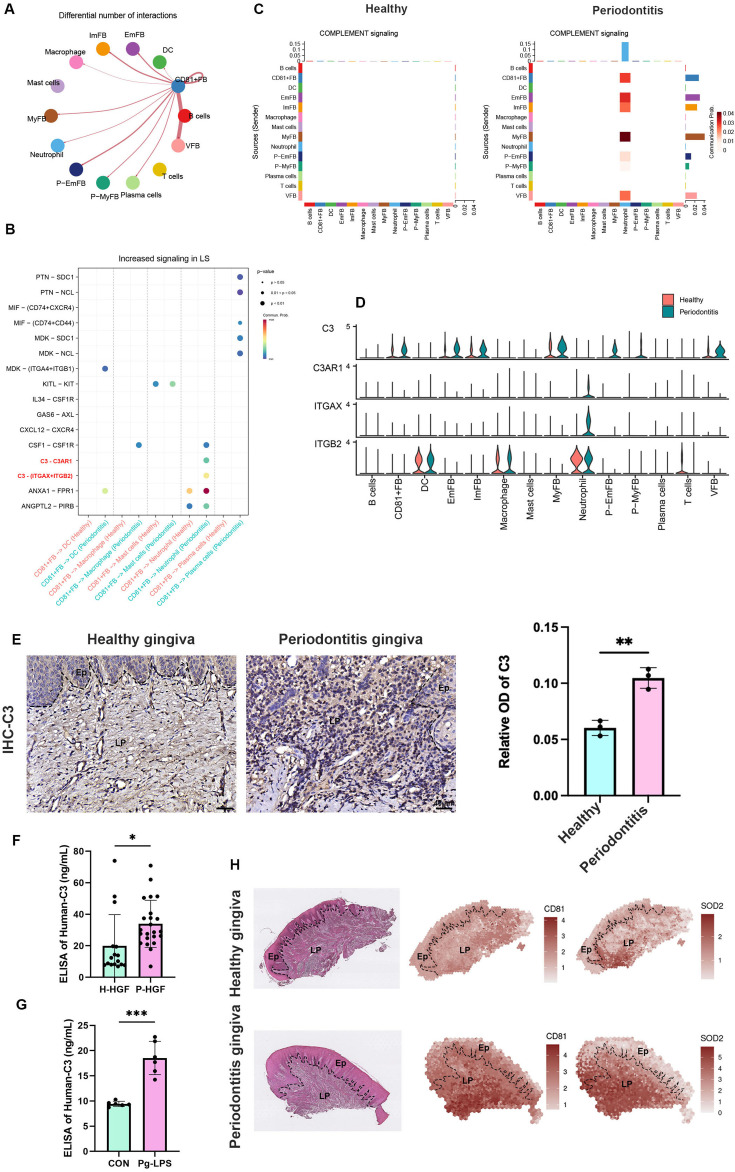


The originally published Figure 5 is shown for reference:

**Figure 7:**
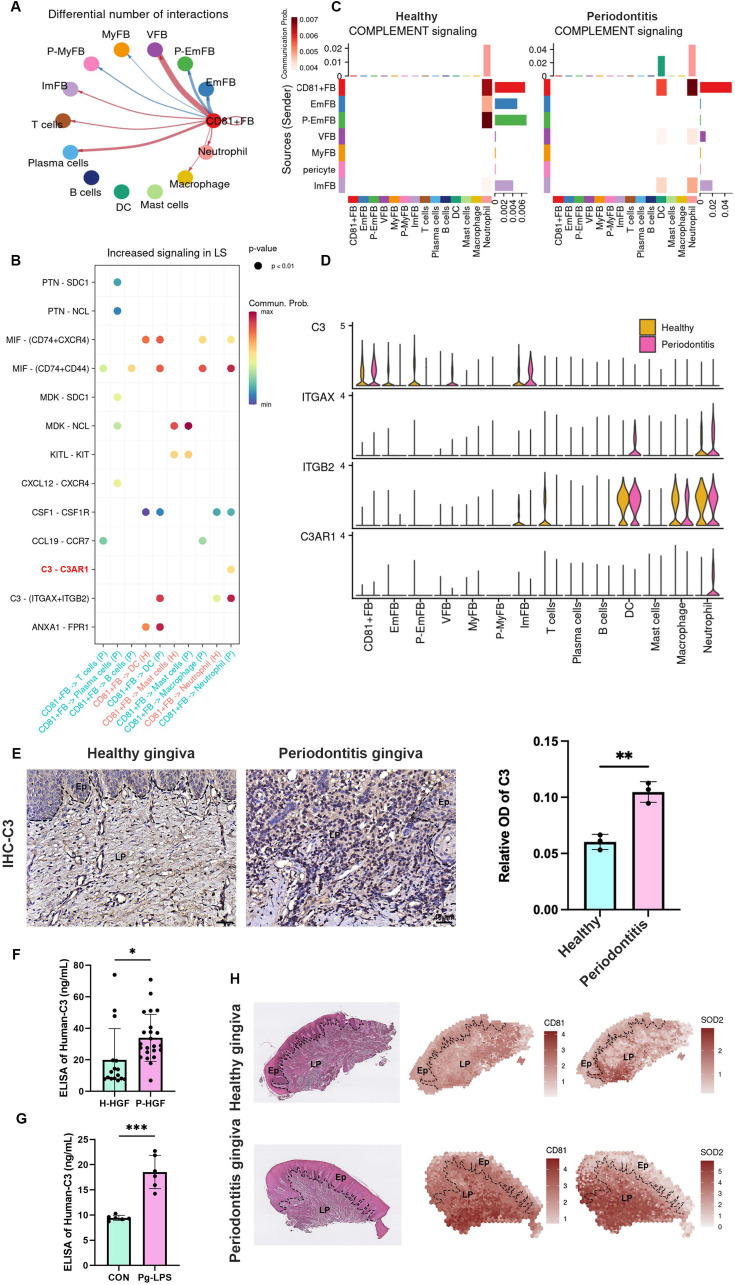


The article has been corrected accordingly.

