# Supplementary figures and images for: CD81+ senescent-like fibroblasts exaggerate inflammation and activate neutrophils via C3/C3aR1 axis in periodontitis
Source: eLife. 2025 Aug 13;13:RP96908. doi: 10.7554/eLife.96908 (PMC12349900; doi:10.7554/eLife.96908)

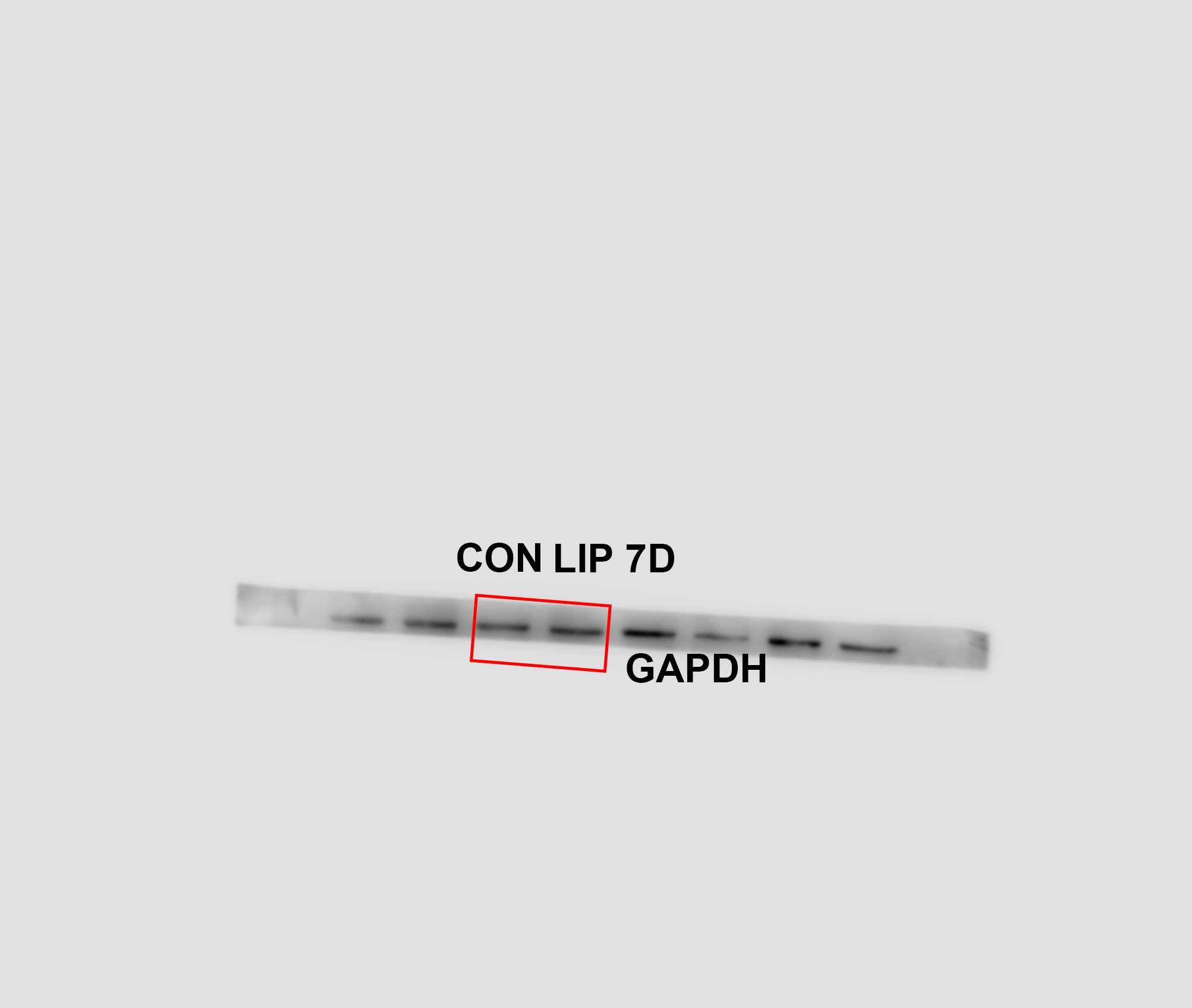

Supplement: Figure 1—source data 1. [file elife-96908-fig1-data1.zip › Figure 1-Source data 1 Uncropped western blots with labeling for panel E/Figure 1E Gapdh with labeling.tif]

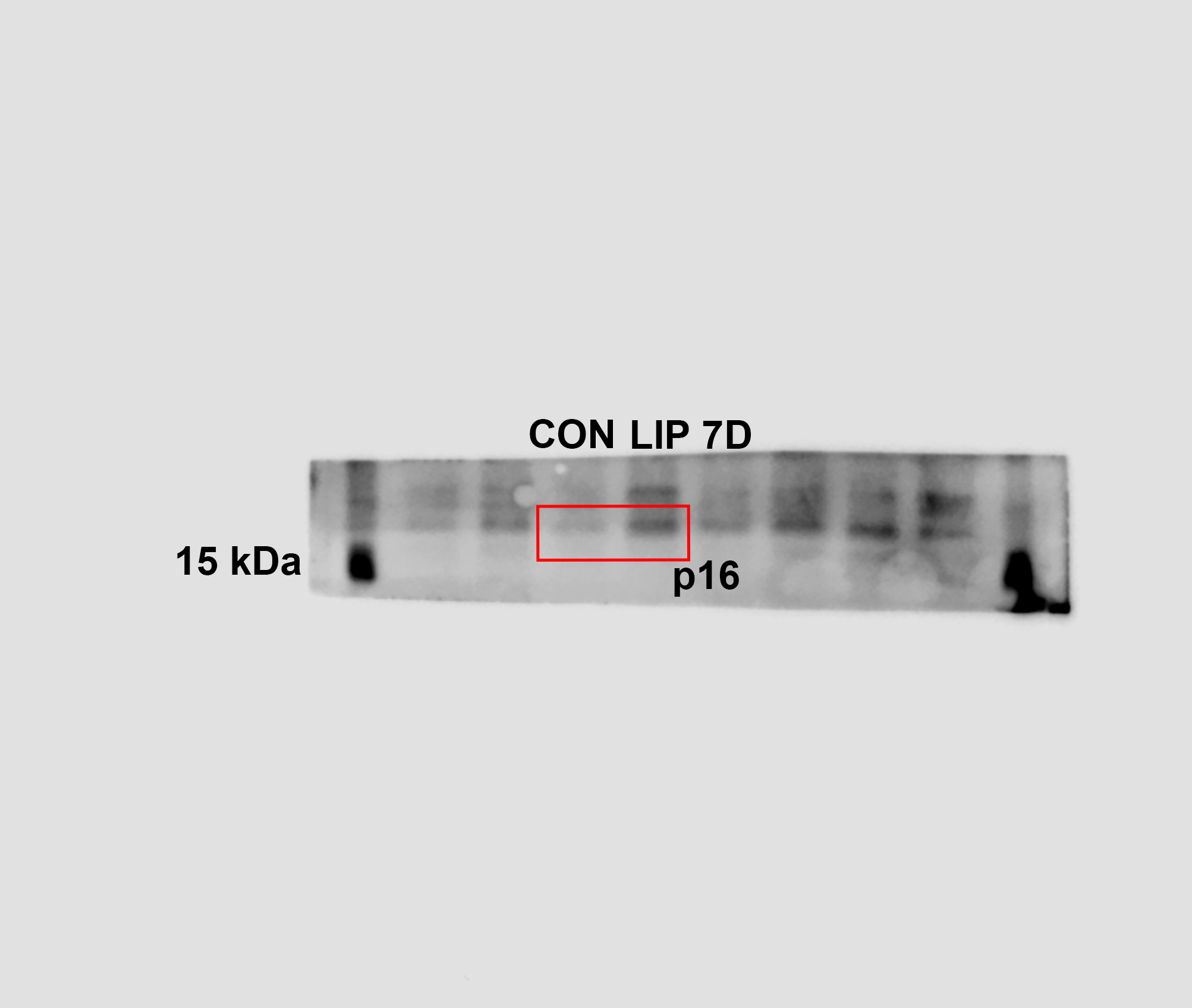

Supplement: Figure 1—source data 1. [file elife-96908-fig1-data1.zip › Figure 1-Source data 1 Uncropped western blots with labeling for panel E/Figure 1E p16 with labeling.tif]

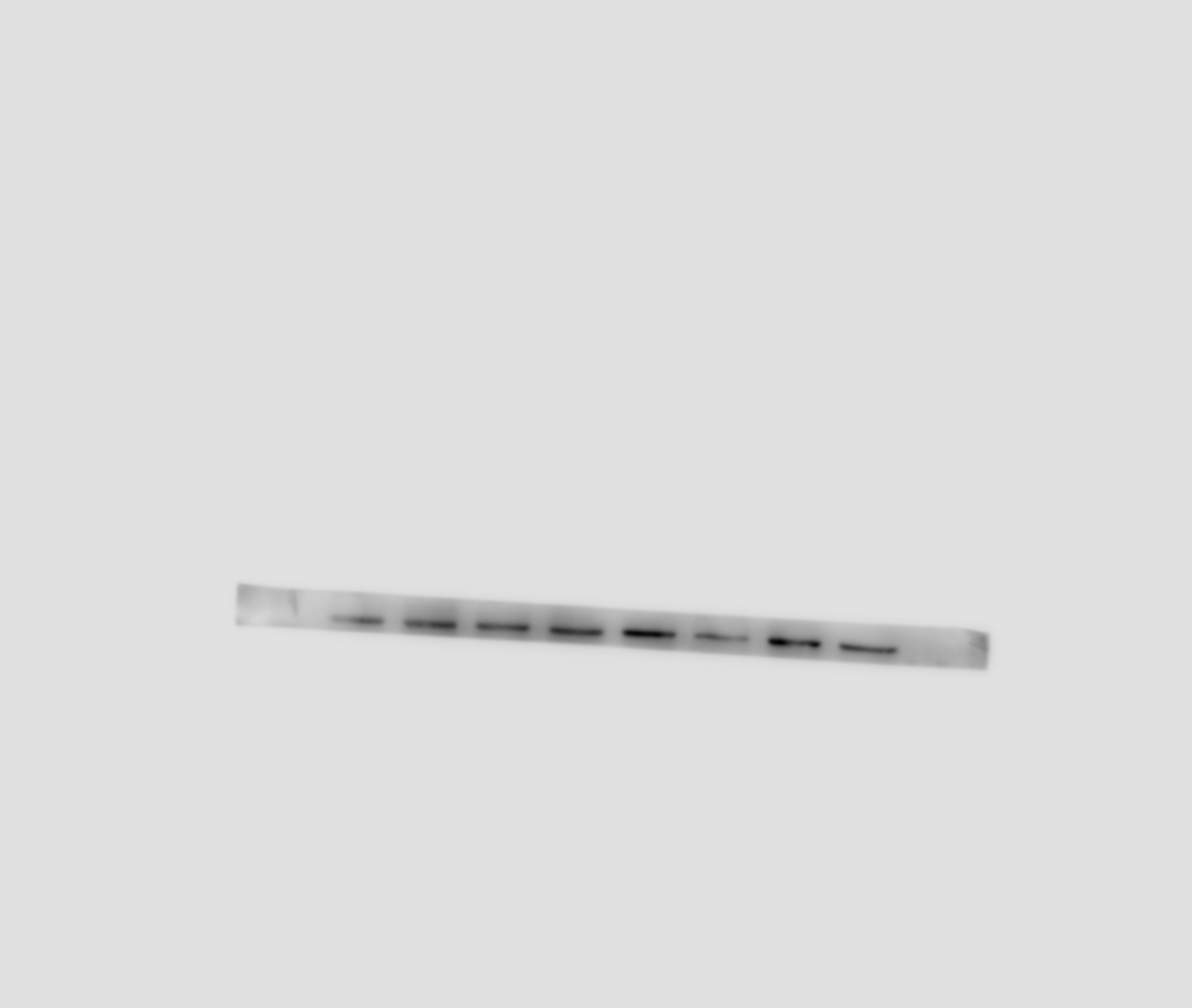

Supplement: Figure 1—source data 2. [file elife-96908-fig1-data2.zip › Figure 1-Source data 2 Original tiff files of western blots for panel E/Figure 1E Gapdh .tif]

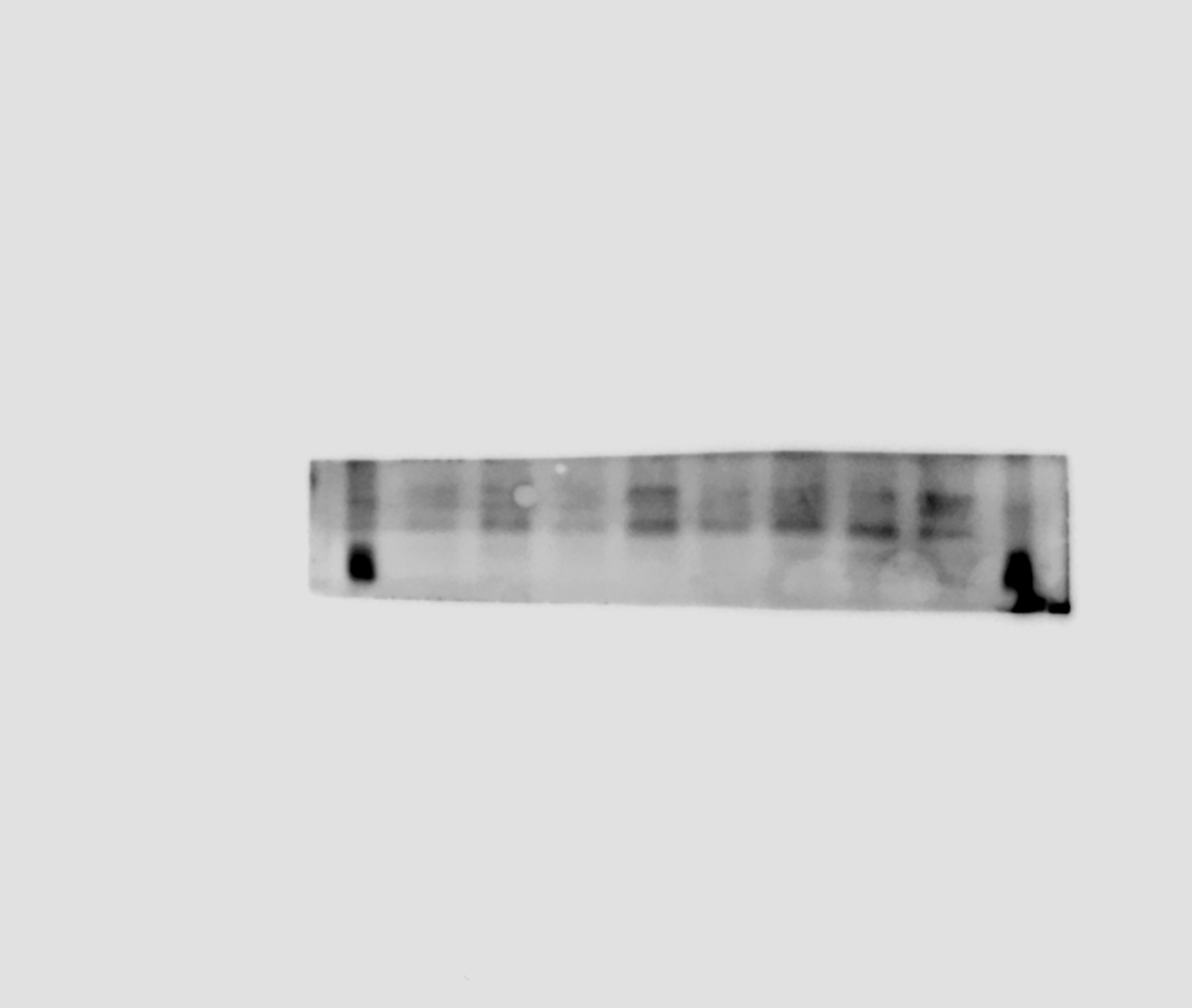

Supplement: Figure 1—source data 2. [file elife-96908-fig1-data2.zip › Figure 1-Source data 2 Original tiff files of western blots for panel E/Figure 1E p16.tif]

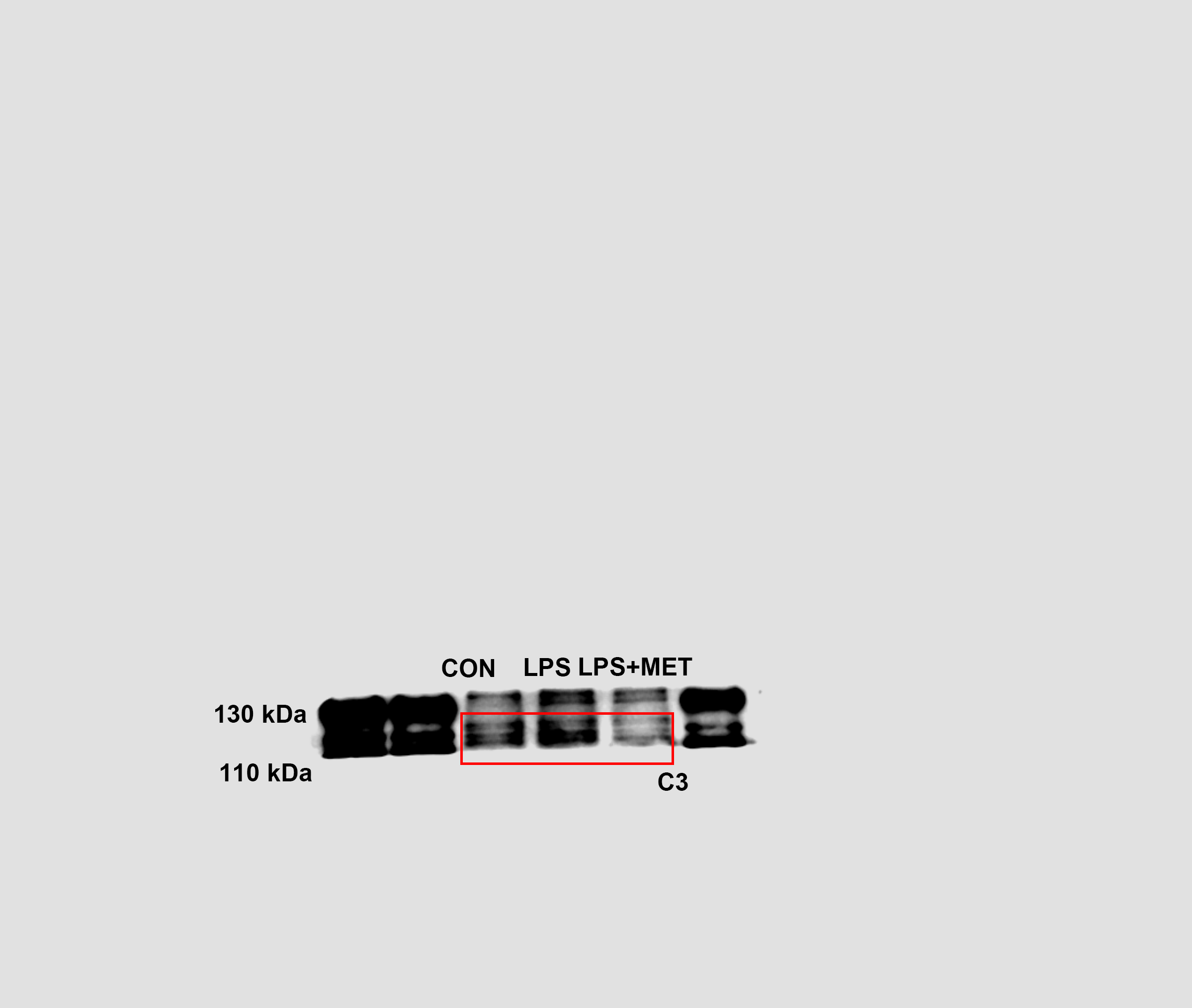

Supplement: Figure 7—figure supplement 2—source data 1. [file elife-96908-fig7-figsupp2-data1.zip › Figure 7-figure supplement 2-Source data 1 Uncropped western blots with labeling for panel D/Figure 7-figure supplement 2 C3 with labeling.tif]

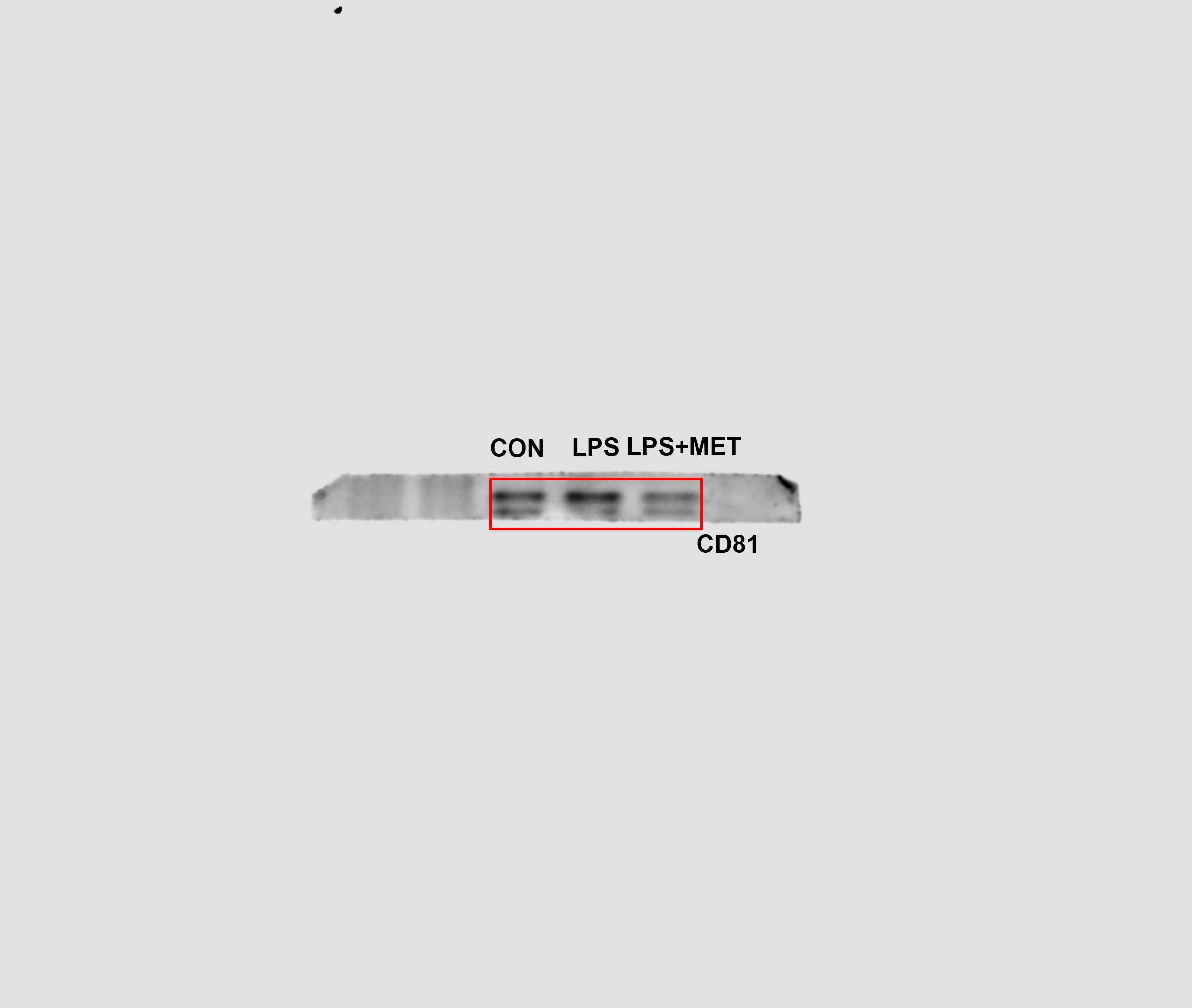

Supplement: Figure 7—figure supplement 2—source data 1. [file elife-96908-fig7-figsupp2-data1.zip › Figure 7-figure supplement 2-Source data 1 Uncropped western blots with labeling for panel D/Figure 7-figure supplement 2 CD81 with labeling.tif]

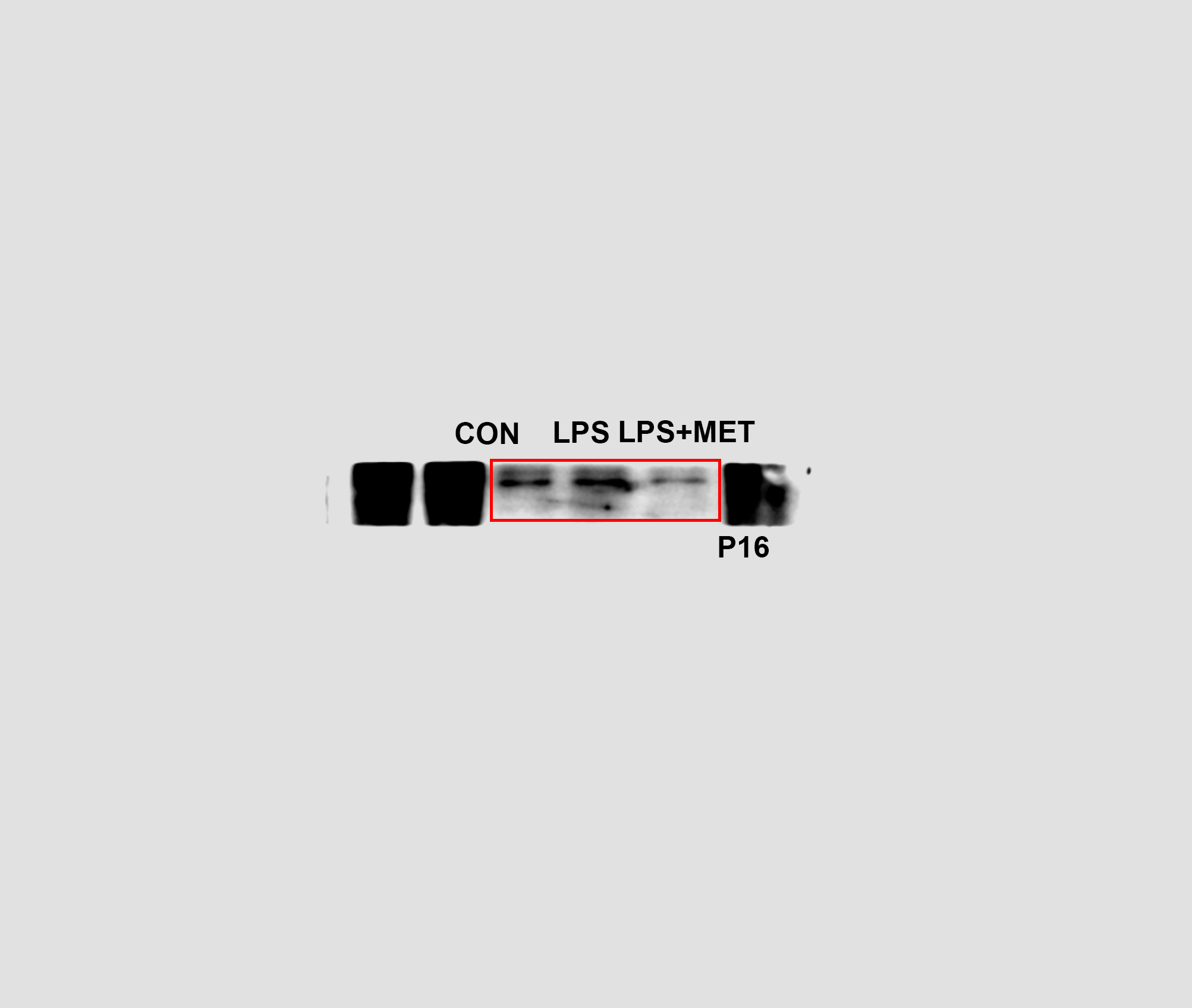

Supplement: Figure 7—figure supplement 2—source data 1. [file elife-96908-fig7-figsupp2-data1.zip › Figure 7-figure supplement 2-Source data 1 Uncropped western blots with labeling for panel D/Figure 7-figure supplement 2 P16 with labeling.tif]

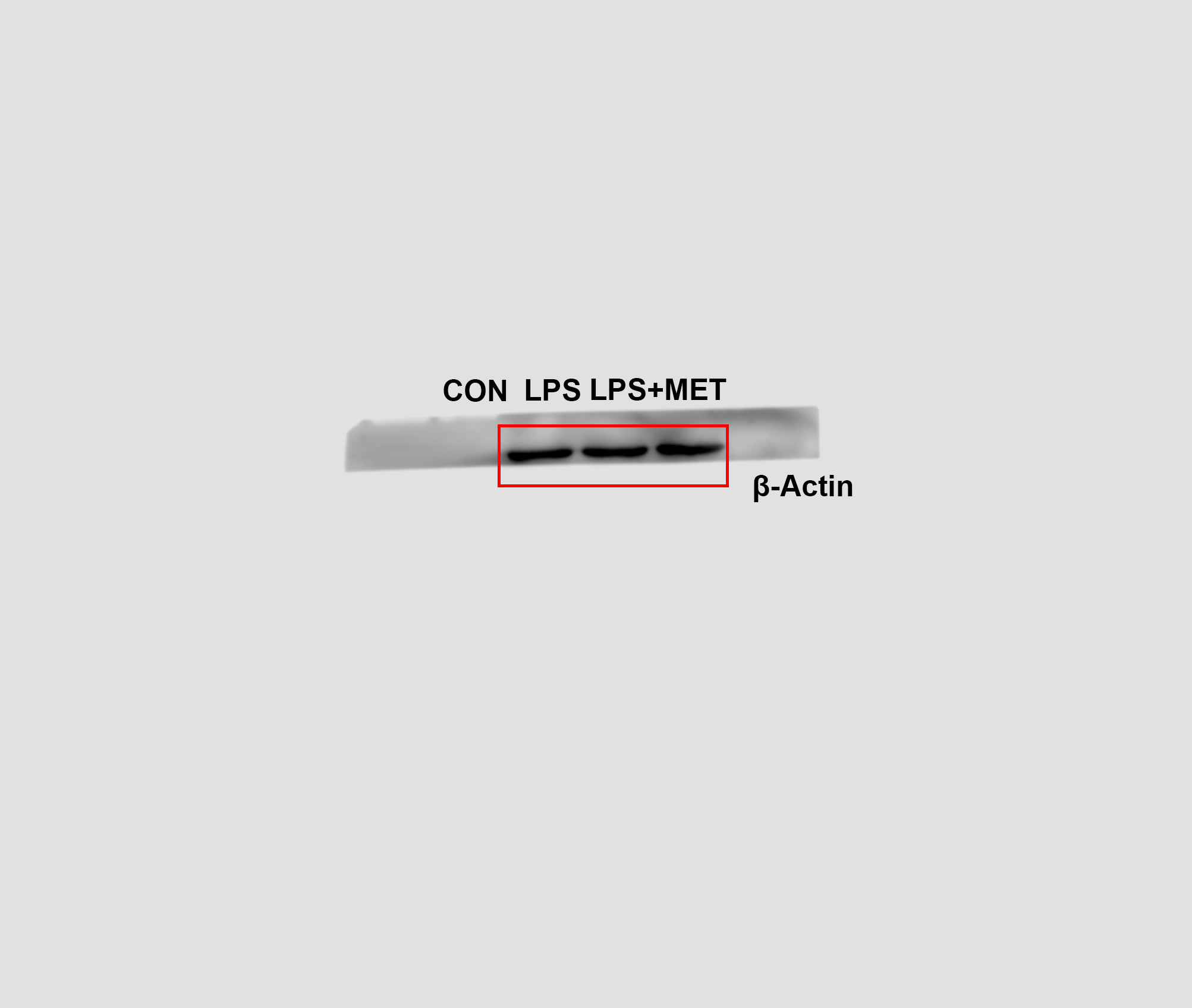

Supplement: Figure 7—figure supplement 2—source data 1. [file elife-96908-fig7-figsupp2-data1.zip › Figure 7-figure supplement 2-Source data 1 Uncropped western blots with labeling for panel D/Figure 7-figure supplement 2 β-ACTIN with labeling.tif]

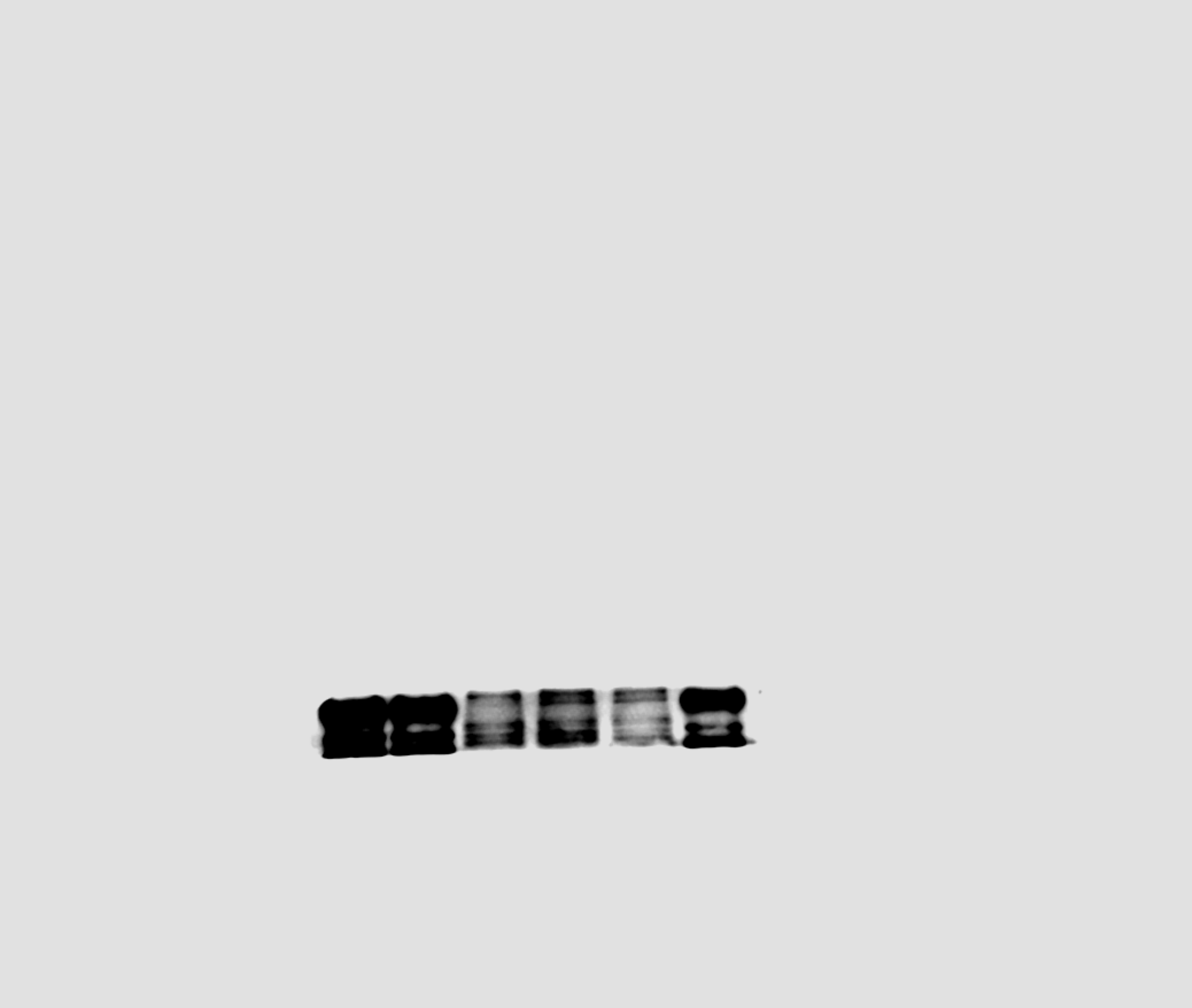

Supplement: Figure 7—figure supplement 2—source data 2. [file elife-96908-fig7-figsupp2-data2.zip › Figure 7-figure supplement 2-Source data 2 Original tiff files of western blots for panel D/Figure 7-figure supplement 2 C3.tif]

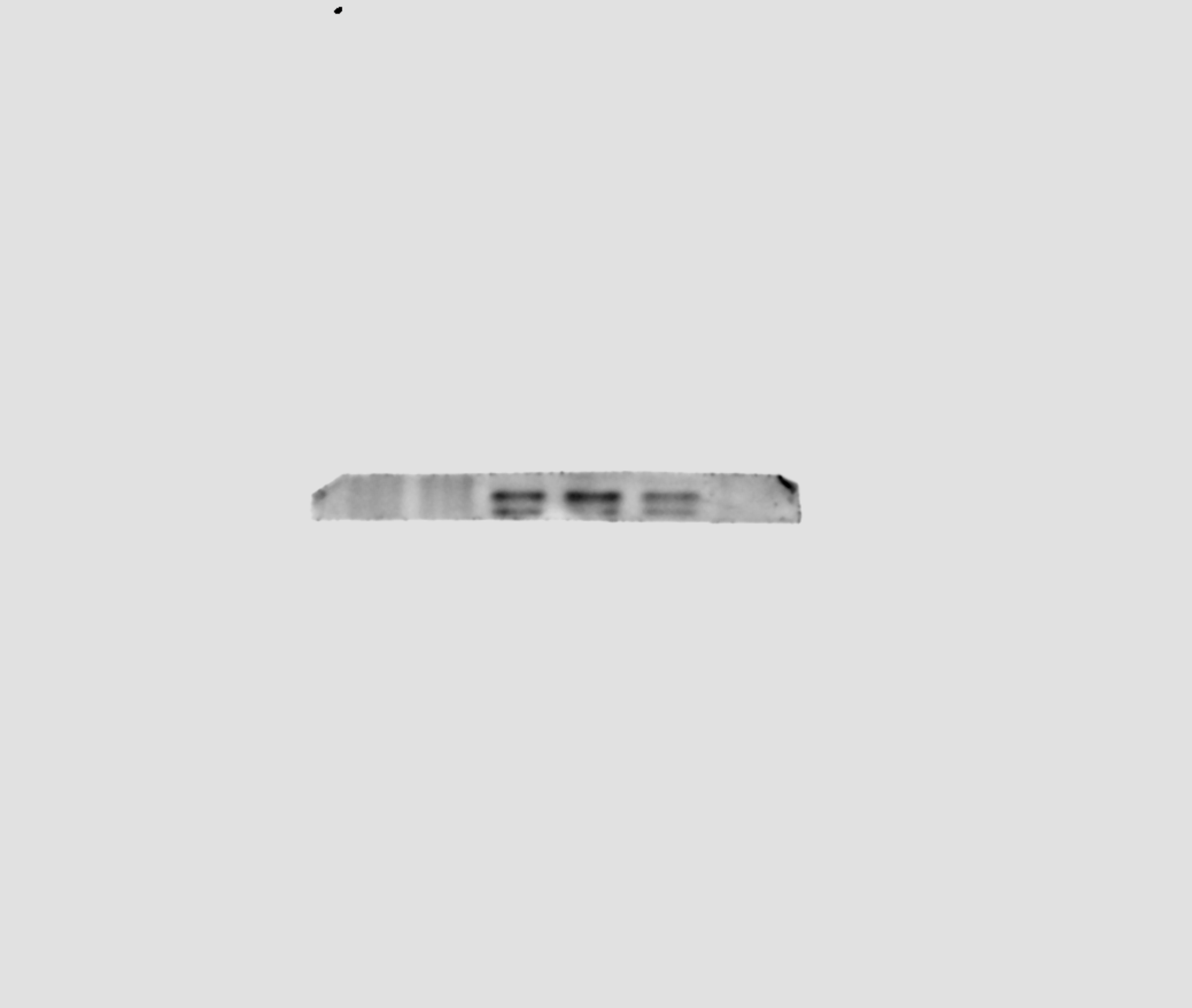

Supplement: Figure 7—figure supplement 2—source data 2. [file elife-96908-fig7-figsupp2-data2.zip › Figure 7-figure supplement 2-Source data 2 Original tiff files of western blots for panel D/Figure 7-figure supplement 2 CD81.tif]

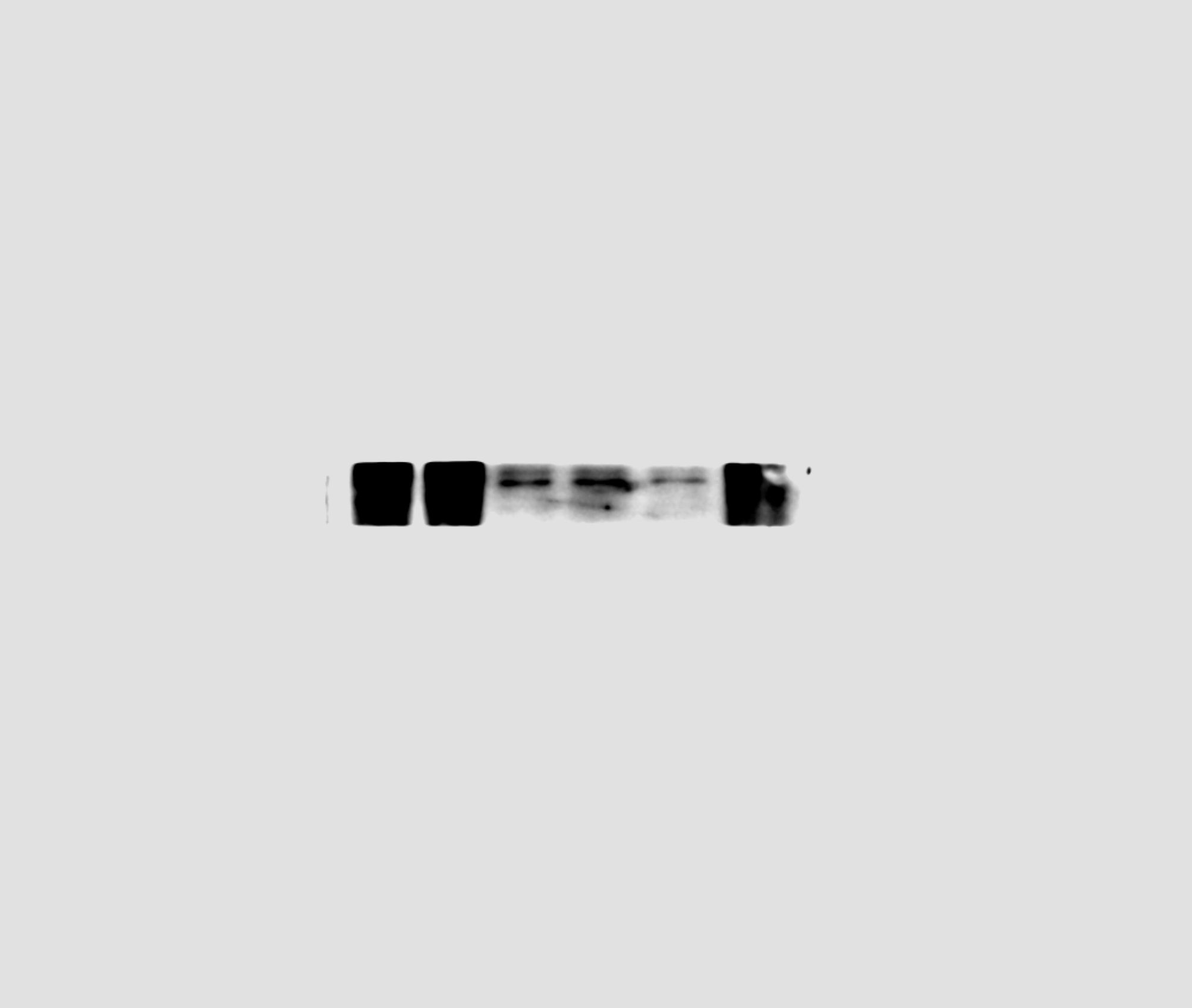

Supplement: Figure 7—figure supplement 2—source data 2. [file elife-96908-fig7-figsupp2-data2.zip › Figure 7-figure supplement 2-Source data 2 Original tiff files of western blots for panel D/Figure 7-figure supplement 2 P16.tif]

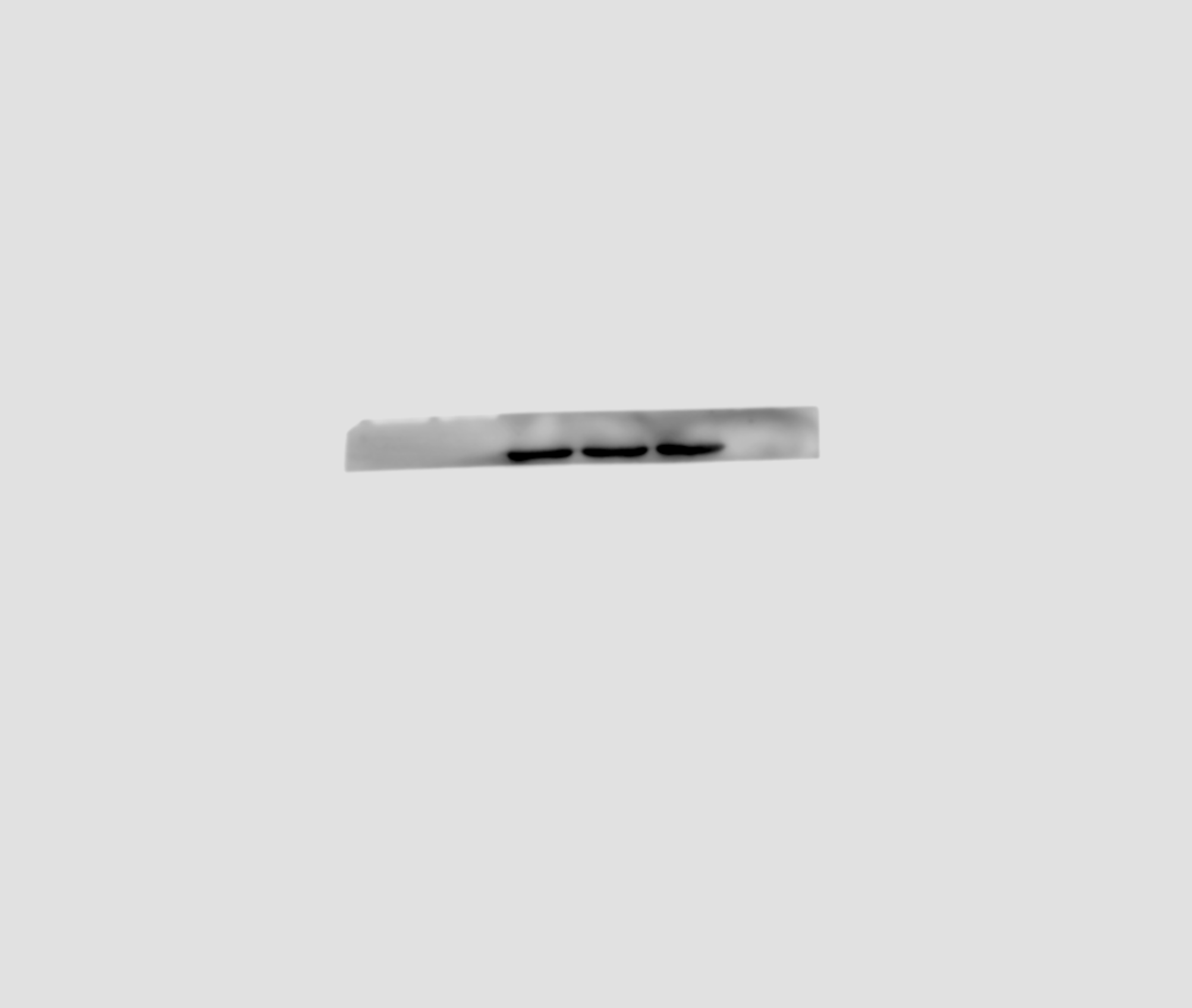

Supplement: Figure 7—figure supplement 2—source data 2. [file elife-96908-fig7-figsupp2-data2.zip › Figure 7-figure supplement 2-Source data 2 Original tiff files of western blots for panel D/Figure 7-figure supplement 2 β-ACTIN.tif]
