# Supplementary material for: CD81+ senescent-like fibroblasts exaggerate inflammation and activate neutrophils via C3/C3aR1 axis in periodontitis
Source: eLife. 2025 Aug 13;13:RP96908. doi: 10.7554/eLife.96908 (PMC12349900; doi:10.7554/eLife.96908)
Supplement: Supplementary file 1. [file elife-96908-supp1.docx]

**CD81^+^ senescent fibroblasts exaggerate inflammation and activate neutrophils via C3/C3aR1 axis in periodontitis**

**Liangliang Fu ^a,1^, Chenghu Yin ^a,1^, Qin Zhao ^a^, Shuling Guo ^a^, Wenjun Shao ^a^, Ting Xia ^a^, Quan Sun ^a^,** **Liangwen Chen ^a^, Jinghan Li ^a^, Min Wang ^a,^ ***, **Haibin Xia ^a,^ *,**

^a^ State Key Laboratory of Oral & Maxillofacial Reconstruction and Regeneration, Key Laboratory of Oral Biomedicine Ministry of Education, Hubei Key Laboratory of Stomatology, School & Hospital of Stomatology, Wuhan University, Wuhan 430079, China; fuliangliang@whu.edu.cn (L.F.); yinchenghu@whu.edu.cn (C.Y.)

^1^ These authors contribute equally to this work.

*****Corresponding author: Min Wang ^a,^ *, Haibin Xia ^a,^ *,

Post address: Department of Oral Implantology, School and Hospital of Stomatology, Wuhan University, 237 Luoyu Road, Wuhan, 430079, Hubei Province, PR China

E-mail addresses: xhaibin@whu.edu.cn (H.X.); 83wangmin@whu.edu.cn (M.W.)

**Declaration of Competing Interest**

The authors declare that they have no known competing financial interests or personal relationships that could have appeared to influence the work reported in this paper.

**Table A. Basic information of included clinical patients**

| **Sample** | **Group** | **Gender** | **Age(year)** | **Usage** |
| --- | --- | --- | --- | --- |
| Patient 1 | Healthy | Male | 21 | Primary cell culture |
| Patient 2 |  | Female | 20 |  |
| Patient 3 |  | Female | 33 | Histological analysis |
| Patient 4 |  | Male | 25 |  |
| Patient 5 |  | Male | 27 |  |
| Patient 6 |  | Male | 28 | Frozen section |
| Patient 7 |  | Male | 22 |  |
| Patient 8 |  | Male | 24 |  |
| Patient 9 | Chronic Periodontitis | Female | 52 | Primary cell culture |
| Patient 10 |  | Male | 62 |  |
| Patient 11 |  | Female | 60 | Histological analysis |
| Patient 12 |  | Female | 50 |  |
| Patient 13 |  | Female | 44 |  |
| Patient 14 |  | Male | 25 | Frozen section |
| Patient 15 |  | Male | 37 |  |
| Patient 16 |  | Female | 28 |  |

**Table B.** Primer sequences for real-time PCR

| Gene | 5'-3' | Primer Sequences (5'- 3') |
| --- | --- | --- |
| Mouse-*p16* | Forward Primer | TGTTGAGGCTAGAGAGGATCTTG |
|  | Reverse Primer | CGAATCTGCACCGTAGTTGAGC |
| Mouse*-p21* | Forward Primer | TCGCTGTCTTGCACTCTGGTGT |
|  | Reverse Primer | CCAATCTGCGCTTGGAGTGATAG |
| Mouse-*Tp53* | Forward Primer | GTCACAGCACATGACGGAGG |
|  | Reverse Primer | TCTTCCAGATGCTCGGGATAC |
| Mouse-*β-actin* | Forward Primer | AGATGACCCAGATCATGTTTGAGA |
|  | Reverse Primer | AGAGCCACCAATCCACACAG |
